# Intraoperative Blood Loss Predicts Local Recurrence After Curative Resection for Stage I–III Colorectal Cancer

**DOI:** 10.1002/wjs.12533

**Published:** 2025-03-15

**Authors:** Kouki Imaoka, Manabu Shimomura, Hiroshi Okuda, Takuya Yano, Wataru Shimizu, Masanori Yoshimitsu, Satoshi Ikeda, Masahiro Nakahara, Mohei Kohyama, Hironori Kobayashi, Yosuke Shimizu, Masatoshi Kochi, Shintaro Akabane, Daisuke Sumitani, Shoichiro Mukai, Yuji Takakura, Yasuyo Ishizaki, Shinya Kodama, Masahiko Fujimori, Sho Ishikawa, Tomohiro Adachi, Minoru Hattori, Hideki Ohdan

**Affiliations:** ^1^ Department of Gastroenterological and Transplant Surgery Graduate School of Biomedical and Health Sciences Hiroshima University Hiroshima Japan; ^2^ Department of Surgery Hiroshima City North Medical Center Asa Citizens Hospital Hiroshima Japan; ^3^ Department of Surgery Hiroshima City Hiroshima Citizens Hospital Hiroshima Japan; ^4^ Department of Gastroenterological Surgery Hiroshima Prefectural Hospital Hiroshima Japan; ^5^ Department of Surgery Onomichi General Hospital Onomichi Japan; ^6^ Department of Surgery Hiroshima General Hospital Hatsukaichi Japan; ^7^ Department of Surgery Hiroshima Memorial Hospital Hiroshima Japan; ^8^ Department of Surgery Kure Medical Center/Chugoku Cancer Center Institute for Clinical Research Kure Japan; ^9^ Department of Surgery National Hospital Organization Higashihiroshima Medical Center Higashihiroshima Japan; ^10^ Department of Surgery JR Hiroshima Hospital Hiroshima Japan; ^11^ Department of Surgery Chugoku Rosai Hospital Kure Japan; ^12^ Department of Surgery Chuden Hospital Hiroshima Japan; ^13^ Department of Surgery National Hospital Organization Hiroshima‐Nishi Medical Center Otake Japan; ^14^ Department of Surgery Yoshida General Hospital Akitakata Japan; ^15^ Department of Surgery Kure City Medical Association Hospital Kure Japan; ^16^ Advanced Medical Skills Training Center Institute of Biomedical and Health Science Hiroshima University Hiroshima Japan

**Keywords:** anastomotic leakage, colorectal cancer, intraoperative blood loss, recurrence pattern

## Abstract

**Background:**

To identify the predictors of local recurrence and distant metastasis after radical surgery for stage I–III colorectal cancer.

**Materials and Methods:**

Patient and tumor characteristics, clinicopathological stages, perioperative factors, and postoperative outcomes, including local and distant recurrence, of patients who underwent primary colorectal resection were evaluated in this multicenter retrospective analysis. Univariate and multivariate regression analyses were performed to identify the risk factors for local and distant recurrences, with a focus on the intraoperative blood loss (IBL) ratio [IBL (mL)/total blood volume (mL)] and postoperative complications.

**Results:**

The risk factors for local and distant recurrence pattern differed. The predictors for local recurrence included perioperative factors, such as the IBL ratio and anastomotic leakage, as well as tumor factors, including pT4, rectal cancer, and poorly differentiated histology, in the multivariate analysis. On the other hand, the predictors for distant recurrence included perioperative factors, such as Clavien–Dindo score ≥ 3, and absence of adjuvant chemotherapy as well as tumor factors including pT stage, pN stage, and rectal cancer. The area under the receiver operating characteristic curve (AUC) for local recurrence in the IBL ratio was 0.745, which was higher than the AUCs for other recurrence patterns in the IBL ratio. Patients with a higher IBL ratio had a higher rate of early local recurrence within 2 years postoperatively (Wilcoxon test and *p* = 0.028).

**Conclusion:**

Reducing IBL and formulating perioperative strategies to prevent anastomotic leakage may help decrease the local recurrence rate and improve prognosis.

## Graphical abstract

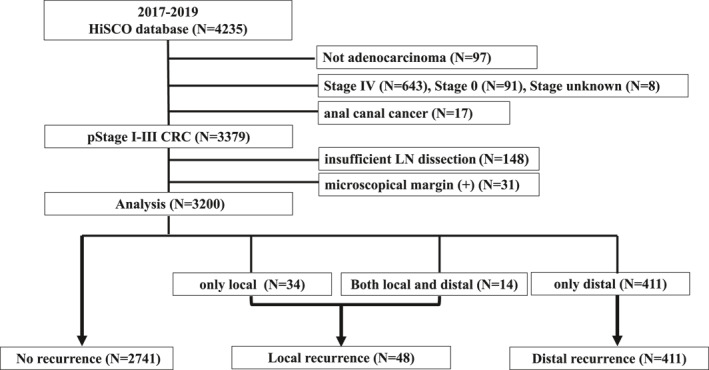

## 1 Introduction

Colorectal cancer (CRC) is the third most common type of cancer affecting both sexes and the leading cause of cancer‐related deaths worldwide [1], with a recurrence rate of approximately 30% after curative resection [2]. The clinicopathological stage of cancer at the time of diagnosis is the most critical predictor of recurrence; however, several other factors also affect patient outcomes [3]. Marked differences have been observed between tumors with different primary tumor locations or histological subtypes in terms of tumor behavior, metastatic pathways, and recurrence patterns, which can be attributed to different embryological and anatomic features [4, 5, 6].

The effect of perioperative factors, such as surgical factors and postoperative complications, on prognosis and recurrence patterns remains controversial. A meta‐analysis reported that greater intraoperative blood loss (IBL) is associated with poor overall survival (OS) and disease‐free survival (DFS) in patients with CRC [7]. Moreover, postoperative complications can worsen the prognosis of CRC and increase the risk of recurrence [8].

Achieving a comprehensive understanding of the risk factors associated with the recurrence patterns plays a crucial role in formulating an optimal postoperative surveillance strategy. However, few studies have evaluated the perioperative risk factors associated with local and distant relapses. Therefore, this retrospective study aimed to assess the perioperative risk factors associated with each recurrence pattern after curative resection of CRC.

## 2 Methods

### 2.1 Patients

**FIGURE 1:**
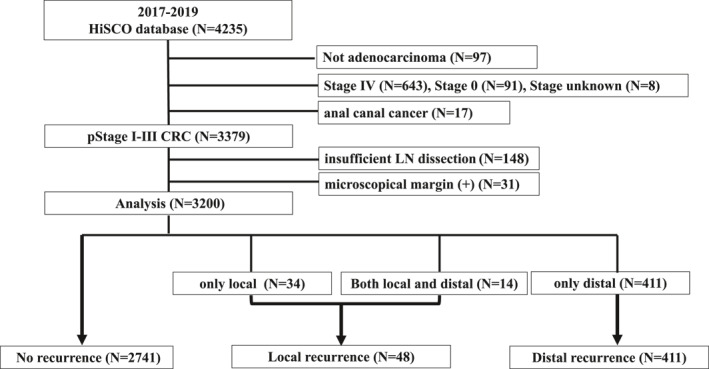
Study consort diagram. Among the 4235 patients, 3200 were included in the analysis. Patients were classified into three groups: no (*n* = 2741; 85.7%), local (*n* = 48; 1.5%), and distant recurrence (*n* = 411; 12.8%) according to the recurrence patterns.

Patients who underwent radical surgery for stage I–III CRC at 15 institutions belonging to the Hiroshima Surgical Study Group of Clinical Oncology (HiSCO) between January 1, 2017, and December 31, 2019 were enrolled in this study. Patients who underwent surgery for nonmalignant diseases or malignancies other than CRC, those with stage IV CRC, those with recurrence, those with inadequate lymph node dissection, and those with microscopical margin positive were excluded. Thus, 3200 patients who underwent radical resection were enrolled in this study (Figure 1). The clinical data of patients, including age, sex, body mass index (BMI), neutrophil‐to‐lymphocyte ratio (NLR) [9], C‐reactive protein/albumin ratio (CAR) [10], pathological findings, tumor location, and perioperative factors, at the time of surgery were extracted. Postoperative complications were defined according to the Clavien–Dindo system [11].

This study was approved by the Institutional Review Board of Hiroshima University Hospital (E2021‐2527). The requirement for obtaining written informed consent was waived owing to the retrospective study design.

### 2.2 Gravimetric Estimation of Intraoperative Blood Loss

We measured IBL using the gravimetric method [12]. In brief, IBL can be estimated by weighing the blood‐soaked surgical materials and subtracting their dry weight. Additionally, the total weight of the collected blood and other fluids (e.g., blood mixed with rinse solution) in the suction container can be measured. By converting 1 g to 1 mL of blood, the total blood loss can be calculated.

### 2.3 Intraoperative Blood Loss Ratio

IBL affects the prognosis and complication rate in patients with CRC; however, a uniform cutoff value for IBL, which ranges from 100 to 450 mL, has not been established [7]. Furthermore, the effect of IBL can vary according to the body weight (BW) of the patients. Therefore, an IBL ratio, calculated using the following equation, was established:

(IBL(mL)/totalbloodvolume[TBV](mL))×100(%)

TBV was calculated using the conventional method as shown below:

TBV(mL)=BW(kg)×{bloodvolume(mL)/kg}.

The following values, as reported by Hilberath et al. [13], were used to adjust for sex: male, 75 mL/kg and female, 65 mL/kg.

### 2.4 Statistical Analysis

Quantitative data are expressed as mean ± standard deviation (SD), whereas categorical variables are summarized as numbers and frequencies. The Student's t‐test or the chi‐squared test were used to compare the mean values of the two groups in univariate analyses. Correlations between IBL and the IBL ratio were tested using the Pearson's correlation coefficient. Kaplan–Meier analysis was performed to determine the OS and the distant or local recurrence rates. These values were compared using the log‐rank or Wilcoxon statistics. Multivariate analyses were performed using the Cox proportional hazards model for variables independently related to distant or local recurrence. Univariate and multivariate Cox regression analyses were performed to assess the association of the following variables with local or distant recurrence: age, sex, BMI, NLR, CAR, CEA, CA19‐9, IBL ratio, transfusion, pT, pN, tumor location, poorly differentiated histology, emergency surgery, surgical approach, postoperative complications (CD ≥ 3), anastomotic leakage (AL), and adjuvant chemotherapy. All variables were included in the multivariate models. The covariates were selected using the backward elimination method with a removal criterion of *p* = 0.05. Statistical differences were determined using Pearson's correlation coefficient. The area under the receiver operating characteristic curve (AUC), homogeneity, discriminatory ability, and Akaike information criteria were evaluated to assess the performance of the prognostic systems. All statistical analyses were performed using the JMP statistical software (JMP 16; SAS Institute Inc., Cary, NC, USA). Statistical significance was set at *p* < 0.05.

## 3 Results

### 3.1 Comparison of Model Suitability

Analysis of the correlation between IBL and the IBL ratio showed a strong correlation (Pearson's *r* = 0.949 and *p* < 0.001). The efficacy of the predictive models, including IBL and the IBL ratio, was evaluated using AUC values, and each model's performance in overall survival (OS), recurrence‐free survival (RFS), and recurrence was compared. For OS, the IBL ratio showed a higher AUC (0.602; 95% CI, 0.571–0.633) than IBL (0.595; 95% CI, 0.563–0.625, *p* = 0.007). Similarly, for RFS, the IBL ratio exhibited a higher AUC (0.590; 95% CI, 0.565–0.615) than IBL (0.585; 95% CI, 0.560–0.610, *p* = 0.032). For recurrence, the AUC of the IBL ratio (0.602; 95% CI, 0.573–0.630) was also higher than that of IBL (0.595; 95% CI, 0.565–0.623, *p* = 0.009). These findings indicate that the IBL ratio may serve as a more reliable prognostic marker than IBL.

### 3.2 Clinicopathological Characteristics

Among the 3200 patients included in the analysis, recurrence occurred in 459 (14.3%). The primary sites of recurrence were as follows: liver metastasis (*n* = 179; 567%), lung metastasis (*n* = 137; 4.3%), peritoneal dissemination (*n* = 103; 3.2%), recurrent extra‐regional lymph nodes (*n* = 55; 1.7%), local recurrence (*n* = 48; 1.5%), bone metastasis (*n* = 12; 0.4%), brain metastasis (*n* = 8; 0.3%), and other metastases (*n* = 15; 0.45%).

The recurrence patterns were as follows: distant recurrence (*n* = 411, 12.8%), local recurrence (*n* = 34, 1.1%), and both local and distant recurrences (*n* = 14, 0.4%). The patients were classified into three groups: no‐recurrence (*n* = 2741; 85.7%), local recurrence (*n* = 48; 1.5%), and distant recurrence (*n* = 411; 12.8%) (Figure 1). Table 1 presents the baseline patient characteristics.

**TABLE 1:** Clinicopathological characteristics.

	No recurrence *N* = 2741	Recurrence *N* = 459				
		Local *N* = 48	Distant *N* = 411	*p*‐value	Local versus no recurrence/distant	Distant versus no recurrence/local
Age (years)	71 ± 12	69 ± 14	71 ± 12	0.6409	0.3640	0.7646
Sex (male/female)	1481/1260	31/17	198/213	0.0252	0.1188	0.0220
BMI (kg/m^2^)	22.6 ± 3.7	21.8 ± 4.4	22.4 ± 3.8	0.1925	0.1262	0.3656
CAR	0.29 ± 1.06	0.75 ± 1.52	0.38 ± 1.21	0.0079	0.0058	0.1922
NLR	3.2 ± 3.3	4.4 ± 4.7	3.6 ± 4.2	0.0057	0.0283	0.0248
pT				< 0.001	< 0.001	< 0.001
T1	623 (22.7%)	2 (4.2%)	19 (4.6%)			
T2	486 (17.7%)	3 (6.3%)	18 (4.4%)			
T3	1271 (46.4%)	20 (41.7%)	239 (58.2%)			
T4	361 (13.2%)	23 (47.9%)	135 (32.9%)			
pN				< 0.001	0.1083	< 0.001
N0	1946 (71.0%)	25 (52.1%)	154 (37.5%)			
N1	612 (22.3%)	18 (37.5%)	152 (37.0%)			
N2	158 (5.8%)	5 (10.4%)	84 (20.4%)			
N3	25 (0.9%)	0 (0.0%)	21 (5.1%)			
pStage				< 0.001	0.0059	< 0.001
1	967 (35.3%)	5 (10.4%)	25 (6.1%)			
2	979 (35.2%)	20 (41.7%)	129 (31.4%)			
3	795 (29.0%)	23 (47.9%)	257 (62.5%)			
CEA (ng/mL)	8.1 ± 28.1	22.0 ± 45.3	13.6 ± 19.6	< 0.001	0.0011	0.0003
CA19‐9 (U/mL)	27.1 ± 219.5	40.7 ± 111.7	55.2 ± 309.4	0.0711	0.7696	0.0235
Location				< 0.001	0.0011	0.0026
Colon	1842 (67.2%)	21 (43.8%)	248 (60.3%)			
Rectum	899 (32.8%)	27 (56.2%)	163 (39.7%)			
Por/muc	211 (7.7%)	10 (20.8%)	51 (11.4%)	< 0.001	0.0015	0.0190
Emergent surgery (+)	178 (6.5%)	9 (18.8%)	41 (10.0%)	< 0.001	0.0016	0.0161
Operative time (min)	244 ± 90	327 ± 128	259 ± 99	< 0.001	< 0.001	0.0057
Blood loss (mL)	91 ± 213	275 ± 503	149 ± 329	< 0.001	< 0.001	< 0.001
Blood loss ratio (%)	2.4 ± 6.5	8.9 ± 23.6	3.8 ± 8.0	< 0.001	< 0.001	< 0.001
Transfusion (+)	75 (2.7%)	5 (10.4%)	21 (5.1%)	< 0.001	0.0038	0.0148
Surgical approach				< 0.001	0.0016	< 0.001
Laparoscopic surgery	2177 (79.4%)	27 (56.3%)	281 (68.4%)			
Robotic surgery	34 (1.2%)	1 (2.1%)	5 (1.2%)			
Open surgery	530 (19.3%)	20 (41.7%)	125 (30.4%)			
CD ≥ 3	183 (6.7%)	6 (12.5%)	54 (13.1%)	< 0.001	0.1960	< 0.001
Anastomotic leakage (+)	86 (3.1%)	5 (10.4%)	11 (2.7%)	0.0143	0.0041	0.5275
Adjuvant chemotherapy (+)	789 (30.6%)	21 (43.8%)	171 (42.3%)	< 0.001	0.0886	< 0.001

Abbreviations: BMI, body mass index; CAR, C‐reactive protein/albumin ratio; CA19‐9, carbohydrate antigen 19–9; CD, Clavien–Dindo; CEA, carcinoembryonic antigen; NLR, neutrophil‐to‐lymphocyte ratio; pN, pathological lymph node; Por/muc, poorly/mucinous; pStage, pathological stage; T, pathological tumor.

Table 1 presents the differences among the three groups stratified according to the recurrence patterns. CAR and NLR were higher in the local recurrence group than those in the no‐recurrence and distant recurrence groups. The percentage of patients with pT4 and rectal cancer was higher in the local recurrence group. In contrast, the percentage of patients with advanced pN stage lesions was higher in the distant recurrence group. The IBL and IBL ratios in the local recurrence group were higher than those in the no‐recurrence and distant recurrence groups (*p* < 0.001). The percentage of patients who underwent transfusion in the local recurrence group was significantly higher than those in the no‐recurrence and distant recurrence groups (*p* < 0.001). The AL rate in the local recurrence group was higher than those in the no‐recurrence and distant recurrence groups (no recurrence, 3.1%; local recurrence, 10.4%; and distant recurrence, 2.7%; *p* = 0.0041). The proportion of patients who received adjuvant chemotherapy in the distant recurrence group was higher than those in the no‐recurrence and local recurrence groups (*p* < 0.001).

### 3.3 Overall Patient Outcomes

**FIGURE 2:**
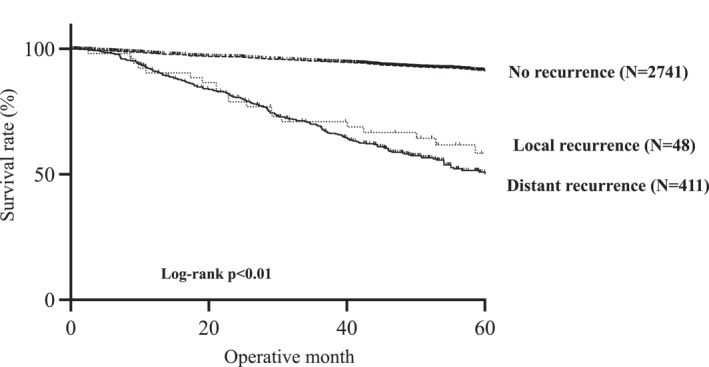
Kaplan–Meier survival curves of the overall survival (OS) among the no, local, and distant recurrence groups. The overall 5‐year survival rates were 91.3%, 57.9%, and 51.8% in the no, local, and distant recurrence groups, respectively (*p* < 0.01).

The overall 5‐year survival rates in the no‐, local, and distant recurrence groups were 91.3%, 57.9%, and 51.8%, respectively (*p* < 0.01 and Figure 2). No significant differences were observed between the distant and local recurrence groups in terms of OS.

### 3.4 The Differences of Clinicopathological Characteristics Between High and Low IBL Ratios

To assess differences in clinicopathologic features by the high and low IBL ratio, the patients were divided into the high IBL ratio group (≥ 2.7%; *N* = 687 [21.5%]) and the low IBL ratio group (< 2.7%; *N* = 2513 [78.5%]) using the average value of the IBL ratio (IBL ratio cutoff value: 2.7%).

Table 2 presents the differences between the two groups. CAR and NLR were higher in the high IBL group than the low IBL group. The percentage of patients with advanced pT stage and pN stage lesions was higher in the high IBL group. Compared to the low IBL group, the high IBL group had a higher rate of emergency surgery and a higher frequency of open surgery. The high IBL group had a significantly higher percentage of patients who received blood transfusions compared to the low IBL group. The rates of postoperative severe complication and AL were significantly higher in the high IBL group than in the low IBL group, and the rates of adjuvant chemotherapy were significantly higher in the high IBL group.

**TABLE 2:** The differences of clinicopathological characteristics between high and low IBL ratios.

	Low IBL ratio (*N* = 2513)	High IBL ratio (*N* = 687)	*p*‐value
Age (years)	70 ± 12	72 ± 12	0.0375
Sex (male/female)	1344/1169	366/321	0.9233
BMI (kg/m^2^)	22.7 ± 3.7	22.3 ± 3.8	0.0290
CAR	0.20 ± 0.74	0.75 ± 1.84	< 0.001
NLR	3.0 ± 3.1	4.3 ± 4.4	< 0.001
pT			< 0.001
T1	582 (23.2%)	62 (9.0%)	
T2	432 (17.2%)	75 (10.9%)	
T3	1154 (45.9%)	376 (54.7%)	
T4	345 (13.7%)	174 (25.3%)	
pN			< 0.001
N0	1719 (68.4%)	406 (59.1%)	
N1	595 (23.7%)	187 (27.2%)	
N2	179 (7.1%)	68 (9.9%)	
N3	20 (0.8%)	26 (3.8%)	
pStage			< 0.001
1	882 (35.1%)	115 (16.7%)	
2	837 (33.4%)	291 (42.4%)	
3	794 (31.6%)	281 (40.9%)	
CEA (ng/mL)	7.4 ± 18.6	15.1 ± 47.3	< 0.001
CA19‐9 (U/mL)	25.2 ± 171.7	52.1 ± 378.2	0.0070
Location			< 0.001
Colon	1707 (67.9%)	404 (58.8%)	
Rectum	806 (32.1%)	283 (41.2%)	
Por/muc	200 (8.0%)	68 (9.9%)	0.1057
Emergent surgery (+)	122 (4.9%)	106 (15.4%)	< 0.001
Operative time (min)	234 ± 79	294 ± 120	< 0.001
Blood loss (mL)	31 ± 28	357 ± 425	< 0.001
Transfusion (+)	25 (1.0%)	76 (11.1%)	< 0.001
Surgical approach			< 0.001
Laparoscopic surgery	2186 (87.0%)	299 (43.5%)	
Robotic surgery	34 (1.4%)	6 (0.9%)	
Open surgery	293 (11.7%)	382 (55.6%)	
CD ≥ 3	152 (6.1%)	91 (13.3%)	< 0.001
Anastomotic leakage (+)	62 (2.5%)	40 (5.8%)	< 0.001
Adjuvant chemotherapy (+)	732 (29.1%)	249 (36.2%)	< 0.001

Abbreviations: BMI, body mass index; CAR, C‐reactive protein/albumin ratio; CA19‐9, carbohydrate antigen 19–9; CEA, carcinoembryonic antigen; CD, Clavien–Dindo; IBL, intraoperative blood loss ratio; NLR, neutrophil‐to‐lymphocyte ratio; pN, pathological lymph node; Por/muc, poorly/mucinous; pStage, pathological stage; T, pathological tumor.

### 3.5 Risk Factors Associated With Local and Distant Recurrence

Multivariate analysis identified pT4 (hazard ratio [HR], 15.2; 95% CI, 3.50–65.8; and *p* = 0.003), IBL ratio (HR, 1.01; 95% CI, 1.00–1.02; and *p* = 0.025), poorly or mucinous differentiated histology (HR, 2.26; 95% CI, 1.07–4.76; and *p* = 0.032), AL (HR, 2.87; 95% CI, 1.11–7.41; and *p* = 0.029), and rectal cancer (HR, 2.58; 95% CI, 1.41–4.71; and *p* = 0.002) as independent predictive factors of local recurrence (Table 3). In contrast, multivariate analysis identified pT stage (pT3: HR, 4.11; 95% CI, 2.52–6.70, *p* < 0.001 aand pT4: HR, 6.50; 95% CI, 3.89–11.2, *p* < 0.001), pN stage (pN1: HR, 2.47; 95% CI, 1.93–3.16, *p* < 0.001; pN2: HR, 4.33; 95% CI, 3.20–5.84, *p* < 0.001; and pN3: HR, 6.66; 95% CI, 4.13–10.7, *p* < 0.001), rectal cancer (HR, 1.31; 95% CI, 1.07–1.61, *p* = 0.009), CD ≥ 3 (HR, 1.85; 95% CI, 1.38–2.48; and *p* < 0.001), and absence of adjuvant chemotherapy (HR, 1.70; 95% CI, 1.37–2.12; and *p* < 0.001) as independent predictive factors of distant recurrence (Table 4). Thus, the risk factors for local and distant recurrence patterns vary, with local recurrence showing strong associations with perioperative factors such as AL and IBL ratio.

**TABLE 3:** Risk factors for local recurrence.

Factors	Univariate			Multivariate		
	HR	95% CI	*p* value	HR	95% CI	*p* value
Age, per 1 year	0.99	0.97–1.02	0.685			
Sex (male)	1.59	0.88–2.88	0.123			
BMI (kg/m^2^), per 1 kg/m^2^	0.93	0.85–1.01	0.067			
CAR, per 1	1.22	1.07–1.39	0.003			
NLR, per 1	1.06	1.01–1.10	0.032			
CEA (ng/mL), per 1 ng/mL	1.00	1.00–1.00	< 0.001			
CA19‐9 (U/mL), per 1 U/mL	1.00	1.00–1.00	0.735			
Tumor location (rectum)	2.48	1.40–4.39	0.002	2.58	1.41–4.71	0.002
pT						
T1	1			1		
T2	1.96	0.33–11.7	0.461	1.71	0.29–10.3	0.556
T3	4.79	1.12–20.5	0.035	4.24	0.99–18.2	0.052
T4	19.1	4.49–80.9	< 0.001	15.2	3.50–65.8	0.003
pN						
N0	1					
N1	2.13	1.16–3.91	0.014			
N2	2.07	0.79–5.42	0.137			
N3	—	—	—			
Emergent surgery (+)	3.47	1.68–7.17	< 0.001			
Surgical approach						
Laparoscopic surgery	1					
Robotic surgery	2.21	0.30–16.3	0.437			
Open surgery	3.06	1.71–5.46	< 0.001			
Intraoperative blood loss ratio (%), per 1%	1.03	1.01–1.03	< 0.001	1.01	1.00–1.02	0.025
Transfusion (+)	4.17	1.65–10.5	0.012			
Por/muc	3.25	1.61–6.54	0.003	2.26	1.07–4.76	0.032
CD ≥ 3	2.01	0.85–4.72	0.144			
Anastomotic leakage (+)	3.73	1.48–9.41	0.005	2.87	1.11–7.41	0.029
Adjuvant chemotherapy (−)	0.66	0.37–1.17	0.152			

Abbreviations: BMI, body mass index; CAR, C‐reactive protein/albumin ratio; CA19‐9, carbohydrate antigen 19–9; CEA, carcinoembryonic antigen; CD, Clavien–Dindo; NLR, neutrophil‐to‐lymphocyte ratio; pN, pathological lymph node; Por/muc, poorly/mucinous; pStage, pathological stage; T, pathological tumor.

**TABLE 4:** Risk factors for distant recurrence.

Factors	Univariate			Multivariate		
	HR	95% CI	*p* value	HR	95% CI	*p* value
Age, per 1 year	1.01	1.00–1.02	0.110			
Sex (male)	0.81	0.67–0.98	0.030			
BMI (kg/m^2^), per 1 kg/m^2^	0.98	0.95–1.01	0.115			
CAR, per 1	1.07	1.00–1.15	0.062			
NLR, per 1	1.04	1.01–1.06	0.004			
CEA (ng/mL), per 1 ng/mL	1.00	1.00–1.00	< 0.001			
CA19‐9 (U/mL), per 1 U/mL	1.00	1.00–1.00	0.073			
Tumor location (rectum)	1.27	1.04–1.55	0.018	1.31	1.07–1.61	0.009
pT						
T1	1			1		
T2	1.24	0.65–2.36	0.517	0.98	0.50–1.93	0.962
T3	5.93	3.72–9.46	< 0.001	4.11	2.52–6.70	< 0.001
T4	11.3	6.99–18.3	< 0.001	6.50	3.89–11.2	< 0.001
pN						
N0	1			1		
N1	2.91	2.32–3.64	< 0.001	2.47	1.93–3.16	< 0.001
N2	5.54	4.25–7.23	< 0.001	4.33	3.20–5.84	< 0.001
N3	8.12	5.14–12.8	< 0.001	6.66	4.13–10.7	< 0.001
Emergent surgery (+)	1.61	1.17–2.23	0.004			
Surgical approach						
Laparoscopic surgery	1					
Robotic surgery	1.07	0.44–2.58	0.888			
Open surgery	1.82	1.47–2.24	< 0.001			
Intraoperative blood loss ratio (%), per 1%	1.02	1.01–1.02	< 0.001			
Transfusion (+)	1.92	1.24–2.98	0.004			
Por/muc	1.52	1.12–2.07	0.007			
CD ≥ 3	2.11	1.59–2.81	< 0.001	1.85	1.38–2.48	< 0.001
Anastomotic leakage (+)	0.88	0.48–1.59	0.664			
Adjuvant chemotherapy (−)	1.45	1.19–1.77	< 0.001	1.70	1.37–2.12	< 0.001

Abbreviations: BMI, body mass index; CAR, C‐reactive protein/albumin ratio; CA19‐9, carbohydrate antigen 19–9; CEA, carcinoembryonic antigen; CD, Clavien–Dindo; NLR, neutrophil‐to‐lymphocyte ratio; pN, pathological lymph node; Por/muc, poorly/mucinous; pStage, pathological stage; T, pathological tumor.

### 3.6 Comparison of the AUC for Recurrence Patterns in IBL Ratios

**FIGURE 3:**
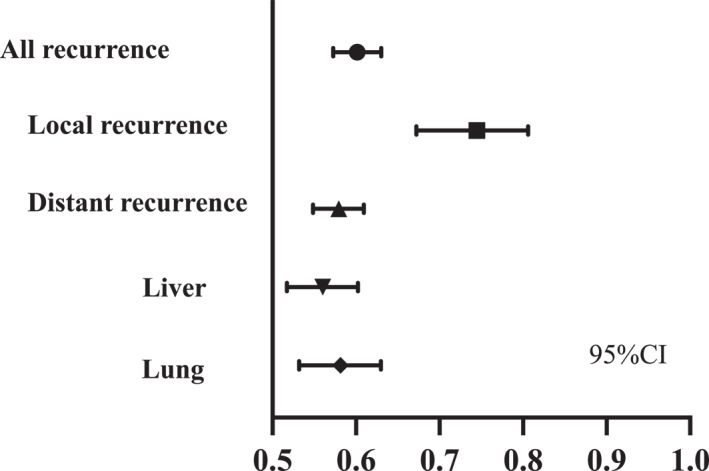
Comparison of the area under the receiver operating characteristic curve (AUC) for recurrence patterns in the intraoperative blood loss ratio. The AUC for all, local, distant, liver, and lung recurrences in the IBL ratio were 0.602 (95% CI, 0.573–0.630), 0.745 (95% CI, 0.672–0.806), 0.579 (95% CI, 0.549–0.609), 0.560 (95% CI, 0.517–0.602), and 0.582 (95% CI, 0.532–0.630), respectively.

The AUC for all, local, distant, liver, and lung recurrences in the IBL ratio were 0.602 (95% CI, 0.573–0.630), 0.745 (95% CI, 0.672–0.806), 0.579 (95% CI, 0.549–0.609), 0.560 (95% CI, 0.517–0.602), and 0.582 (95% CI, 0.532–0.630), respectively (Figure 3). Thus, the IBL ratio may be a useful marker for predicting local recurrence patterns.

### 3.7 Correlation Between Early Local Recurrence and IBL Ratios

**FIGURE 4:**
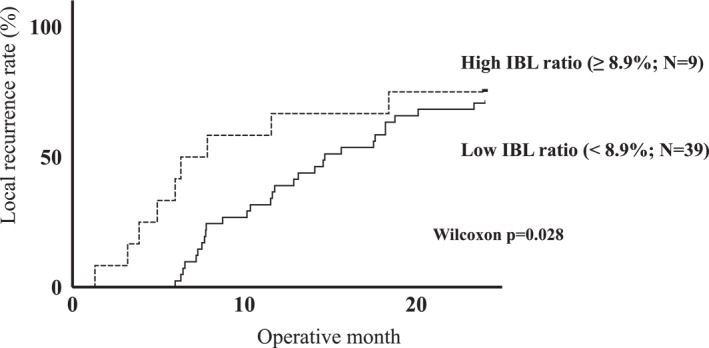
The relationship between the early local recurrence and intraoperative blood loss ratio. Kaplan–Meier estimated survival curve analysis showed significantly worse early recurrence in the high intraoperative blood loss (IBL) ratio group compared with that in the low IBL ratio group (Wilcoxon test and *p* = 0.028).

The association between the IBL ratio and early local recurrence within 2 years of surgery in cases of local recurrence was assessed. The patients in the local recurrence group were divided into the high IBL ratio (≥ 8.9%; *N* = 9 [18.8%]) and low IBL ratio (< 8.9%; *N* = 39 [81.2%]) groups using the average value of the IBL ratio in the local recurrence group (IBL ratio cutoff: 8.9%). Table 5 presents the clinicopathological characteristics of the two groups. No significant differences were observed between the two groups in terms of patient or tumor characteristics. The high IBL ratio group had a lower laparoscopic surgery rate, longer operative time, and higher transfusion rate. Kaplan–Meier estimated survival curve analysis revealed significantly worse early recurrence in the high IBL ratio group compared with that in the low IBL ratio group (Wilcoxon test and *p* = 0.028) (Figure 4). Thus, a higher IBL ratio may facilitate the early detection of local recurrence or contribute to the speed of tumor growth.

**TABLE 5:** Patient characteristics in the local recurrence group.

	Low blood loss ratio (*N* = 39)	High blood loss ratio (*N* = 9)	*p*‐value
Age (years)	69 ± 15	71 ± 9	0.5955
Sex (male/female)	26/13	5/4	0.5298
BMI (kg/m^2^)	22.0 ± 4.2	20.6 ± 5.2	0.3873
CAR	0.61 ± 1.30	1.32 ± 2.24	0.2150
NLR	4.3 ± 5.1	4.6 ± 2.9	0.8528
pT			0.5416
T1	2 (5.1%)	0 (0.0%)	
T2	3 (7.7%)	0 (0.0%)	
T3	17 (43.6%)	3 (33.3%)	
T4	17 (43.6%)	6 (66.7%)	
pN			0.873
N0	21 (53.9%)	4 (44.4%)	
N1	14 (35.9%)	4 (44.4%)	
N2	4 (10.3%)	1 (11.1%)	
N3	0 (0.0%)	0 (0.0%)	
pStage			0.5196
1	5 (12.8%)	0 (0.0%)	
2	16 (41.0%)	4 (44.4%)	
3	18 (46.2%)	5 (5.6%)	
CEA (ng/mL)	23.7 ± 49.7	14.3 ± 15.5	0.5820
CA19‐9 (U/mL)	28.3 ± 61.0	94.5 ± 227.3	0.1100
Location			0.4846
Colon	18 (46.2%)	3 (33.3%)	
Rectum	21 (53.8%)	6 (66.7%)	
Por/muc	7 (17.9%)	3 (33.3%)	0.3056
Emergent surgery (+)	7 (18.0%)	2 (22.2%)	0.7672
Surgical approach			0.0503
Laparoscopic surgery	25 (64.1%)	2 (22.2%)	
Robotic surgery	1 (2.6%)	0 (0.0%)	
Open surgery	13 (33.3%)	7 (77.8%)	
Operative time (min)	301 ± 134	386 ± 75	0.0750
Intraoperative blood loss (mL)	113 ± 96	975 ± 876	< 0.001
Transfusion (+)	1 (2.6%)	4 (44.4%)	< 0.001
Hospital stays (days)	19 ± 12	32 ± 19	0.0192
CD ≥ 3	4 (10.3%)	2 (22.2%)	0.3279
Anastomotic leakage (+)	5 (12.2%)	1 (11.1%)	0.8888
Adjuvant chemotherapy (+)	19 (48.7%)	2 (22.2%)	0.1487

Abbreviations: BMI, body mass index; CAR, C‐reactive protein/albumin ratio; CA19‐9, carbohydrate antigen 19–9; CEA, carcinoembryonic antigen; CD, Clavien–Dindo; NLR, neutrophil‐to‐lymphocyte ratio; pN, pathological lymph node; Por/muc, poorly/mucinous; pStage, pathological stage; T, pathological tumor.

## 4 Discussion

This retrospective analysis of a large and representative HiSCO database aimed to identify the risk factors for local and distant recurrence. Distant recurrence showed significant associations with the clinicopathological stage at the time of diagnosis, rectal cancer, absence of adjuvant chemotherapy, and severe complications (CD ≥ 3). In contrast, local recurrence showed associations with perioperative factors (the IBL ratio and AL) and tumor‐related factors (such as pT4, rectal cancer, and poorly differentiated histology). A notable strength of this study in comparison with previous reports is the inclusion of comprehensive background factors, including detailed patient information and tumor‐related factors. These factors revealed an association between IBL and local recurrence, especially early local recurrence within 2 years postoperatively.

IBL adversely affects the prognosis of patients with CRC [7]. An increase in IBL may lead to a higher incidence of postoperative complications and slower perioperative recovery from surgical intervention, resulting in delayed AC initiation. A previous study highlighted the predictors of local recurrence and remote metastasis of mucinous adenocarcinoma after surgery and revealed that intraoperative transfusion related to bleeding is a significant predictor of local recurrence [14]; however, potential explanations for this mechanism remain unclear. Advanced T stage and associated infiltrative tumor growth can make the performance of high‐quality total mesorectal or complete mesocolic excision difficult, potentially increasing IBL. Furthermore, increased IBL may elevate the risk of intraoperative tumor spillage, which could result in local recurrence. Notably, 30%–40% of patients with metastatic CRC exhibited ≥3 circulating tumor cells (CTC)/7.5 mL of blood [15]. The detection rate of CTC varies significantly according to the stage of CRC. The detection rate is notably low for Stage I CRC, whereas it is significantly higher for Stage III CRC [16]. Thus, an increase in the IBL may result in a higher number of CTCs in the hemorrhaged blood, leading to an increase in the viability and dissemination of tumor cells within the resection area. Increased IBL may lead to tissue hypoperfusion and insufficient oxygenation. These conditions suppress the production of mitogen‐induced lymphocytes [17] and interleukin‐2 (IL‐2) [18], thereby impairing the antitumor immune response. Furthermore, this condition also elevates the expression of vascular endothelial growth factors, thereby promoting tumor angiogenesis [19]. Hypoxia contributes to genomic instability, resulting in various genetic alterations that lead to the creation of a more aggressive phenotype in residual tumor cells [20]. These mechanisms may support the findings of the present study that the IBL ratio affects the recurrence patterns of CRC differently and that higher tumor exposure caused by greater IBL within the surgical area may lead to faster tumor growth and earlier detection of local recurrence.

The effect of AL on the recurrence patterns of CRC is controversial [21, 22, 23, 24]. AL had a negative effect on the survival of patients with stages II and III colon cancer and stages III and IV rectal cancer in a retrospective Dutch population‐based study; however, it showed no independent association with disease recurrence [21]. AL shows significant associations with increased rates of local and distant recurrence after colon cancer surgery [22, 23]. The mechanism via which AL enhances tumor recurrence remains uncertain; however, AL can potentially promote viable tumor cells to retain their oncological competence via immunosuppression [23, 25]. Furthermore, the local inflammatory response associated with AL can facilitate local recurrence of cancer [26]. Several inflammatory mediators, including pro‐inflammatory ILs (IL‐1 and IL‐6), are involved in tumor growth, survival, prevention of apoptosis, progression to metastasis, and resistance to drug therapy [24, 27, 28]. The findings of the present study revealed that AL showed a strong association with local recurrence but not with distant recurrence. In contrast, CD ≥ 3 showed a strong association with distant recurrence but not with local recurrence. Nevertheless, the effect of AL on the clinical outcomes of CRC surgery should be interpreted with caution as the severity of AL was not evaluated in the present study. A previous study showed that symptomatic and asymptomatic AL had different effects on the oncological outcomes after rectal resection [25]. Further studies are required to clarify the prognostic impact of AL by considering the degree of inflammation caused by AL, either systemically or locally. A confluence of factors encompassing oncologic characteristics that predispose patients to intraperitoneal seeding, such as pT4 and poorly differentiated histology, the degree of IBL (which may influence tumor exposure), and the high inflammatory status associated with AL (where tumor cells are prone to survival and proliferation), may contribute to an increase in local recurrence.

This study had some limitations that should be considered when interpreting the findings. First, this was a retrospective cohort study, which may have resulted in the potential selection and detection bias. Second, the low incidence of local recurrence in the study cohort may have influenced the findings of the present study. Furthermore, information on the RAS and RAF gene mutation status in the tumors was lacking. Further prospective and national studies must be conducted to overcome these limitations.

In conclusion, this study identified different risk factors for local and distant recurrences after radical CRC surgery. Perioperative risk factors, including the IBL ratio and AL, which were significantly associated with local recurrence, were specifically analyzed. Severe postoperative complications were associated with distant recurrence. Surgeons should aim to reduce IBL and prevent perioperative AL to decrease the local recurrence rate and improve the prognosis.

## Author Contributions

**Kouki Imaoka:** conceptualization, formal analysis, investigation, methodology, project administration, writing – original draft, writing – review and editing. **Manabu Shimomura:** conceptualization, data curation, formal analysis, funding acquisition, investigation, methodology, project administration, writing – review and editing. **Hiroshi Okuda:** conceptualization, data curation, formal analysis, investigation, methodology, project administration, writing – review and editing. **Takuya Yano:** conceptualization, data curation, investigation, methodology, writing – review and editing. **Wataru Shimizu:** data curation, investigation, writing – review and editing. **Masanori Yoshimitsu:** data curation, investigation, writing – review and editing. **Satoshi Ikeda:** data curation, investigation, writing – review and editing. **Masahiro Nakahara:** data curation, investigation, writing – review and editing. **Mohei Kohyama:** data curation, investigation, writing – review and editing. **Hironori Kobayashi:** data curation, investigation, writing – review and editing. **Yosuke Shimizu:** data curation, investigation, writing – review and editing. **Masatoshi Kochi:** data curation, investigation, writing – review and editing. **Shintaro Akabane:** data curation, investigation, writing – review and editing. **Daisuke Sumitani:** data curation, investigation, writing – review and editing. **Shoichiro Mukai:** data curation, investigation, writing – review and editing. **Yuji Takakura:** data curation, investigation, writing – review and editing. **Yasuyo Ishizaki:** data curation, investigation, writing – review and editing. **Shinya Kodama:** data curation, investigation, writing – review and editing. **Masahiko Fujimori:** data curation, investigation, writing – review and editing. **Sho Ishikawa:** data curation, investigation, project administration. **Tomohiro Adachi:** data curation, investigation, project administration, writing – review and editing. **Minoru Hattori:** formal analysis, writing – review and editing. **Hideki Ohdan:** conceptualization, data curation, formal analysis, investigation, methodology, project administration, supervision, writing – review and editing.

## Ethics Statement

This study was conducted in accordance with the tenets of the Declaration of Helsinki. This study was authorized in advance by the institutional review board of Hiroshima University Hospital (registration number of the study: E2021‐2527).

## Consent

The requirement for written informed consent was waived because of the retrospective design of this study.

## Conflicts of Interest

The authors declare no conflicts of interest.

## Data Availability

Research data are not available.
